# Association between foot posture and tibiofemoral contact forces during barefoot walking in patients with knee osteoarthritis

**DOI:** 10.1186/s12891-022-05624-y

**Published:** 2022-07-12

**Authors:** Takanari Kubo, Daisuke Uritani, Shinya Ogaya, Shunsuke Kita, Takahiko Fukumoto, Tadashi Fujii, Yusuke Inagaki, Yasuhito Tanaka, Hidetaka Imagita

**Affiliations:** 1 Graduate School of Health Sciences, Kio University, 4-2-2 Umaminaka, Koryo-cho, Kitakatsuragi-gun, Nara, 635-0832 Japan; 2 Department of Rehabilitation, Osaka Kawasaki Rehabilitation University, 158 Mizuma, Kaizuka, Osaka 597-0104 Japan; 3 Department of Physical Medicine and Rehabilitation, Kansai Medical University, 2-5-1 Shinmachi, Hirakata, Osaka 573-1010 Japan; 4 Department of Physical Therapy, School of Health and Social Services, Saitama Prefectural University, 820 Sannomiya, Koshigaya-shi, Saitama, 343-8540 Japan; 5 Soka Orthopedics Internal Medicine, 1-1-18 Chuo, Soka, Saitama 340-0016 Japan; 6 Department of Orthopaedic Surgery, Kashiba Asahigaoka Hospital, 839 Kaminaka, Kashiba, Nara Japan; 7 Department of Orthopaedic Surgery, Nara Medical University, Shijocho 840, Kashihara, Nara 634-8522 Japan

**Keywords:** Tibiofemoral contact force, Foot, Walking, Musculoskeletal model

## Abstract

**Background:**

Accumulating evidence indicates that abnormal foot posture are risk factors for knee osteoarthritis (OA). However, the relationship between foot posture and tibiofemoral contact force (CF) during habitual weight-bearing activities remains unclear. This study aimed to determine the association between tibiofemoral CF and foot posture while walking.

**Methods:**

In total, 18 patients with knee OA and 18 healthy individuals participated in this cross-sectional study. Foot parameters were evaluated by Foot Posture Index (FPI), Staheli Arch Index (SAI), hallux valgus angle, calcaneus inverted angle relative to the floor as a static rearfoot posture, navicular height, and toe grip strength. In addition, all participants underwent kinetic and kinematic measurements during a self-selected speed gait. The measurement device used was the three-dimensional motion analysis system with a sampling rate of 120 Hz. The musculoskeletal model, which has 92 Hill-type muscle–tendon units with 23 degrees of freedom, was used to calculate tibiofemoral CF. Partial correlations was used to investigate the association between foot parameters and total, medial, and lateral tibiofemoral CF of the first and second peaks while controlling for gait speed.

**Results:**

A significant negative correlation was observed between Walking SAI and first peak medial tibiofemoral CF in control participants (*r* = -0.505, *p* = 0.039). SAI was also significantly positively correlated with first peak medial tibiofemoral CF in patients with knee OA (*r* = 0.482, *p* = 0.042).

**Conclusions:**

Our findings revealed a correlation between the medial first peak tibiofemoral CF and the SAI. This study indicates that people with knee OA and flatfoot have excessive first medial tibiofemoral CF during walking.

**Supplementary Information:**

The online version contains supplementary material available at 10.1186/s12891-022-05624-y.

## Background

Knee osteoarthritis (KOA), a highly prevalent disease in older adults, leads to chronic pain, stiffness, and disability. Various factors contribute toward dynamic mechanical loading of the knee joint during walking that can lead to joint pain [1], cartilage damage [2], and bone deformities [3]. Further, the development and progression of KOA is affected by excessive rotational and/or contact loading [2, 3]. Although radiographic KOA is rare in Japanese people aged < 40 years, its prevalence among adults aged > 40 years has been demonstrated to be 42.6% in men and 62.4% in women [4].

Many studies have assessed the association between tibiofemoral joint (TFJ) contact loading by knee adduction moment (KAM) and knee function or alignment. For example, Winby et al. [5] demonstrated that KAM during walking was influenced by knee-spanning muscle contractions in healthy individuals, and Sharma et al. [6] found a negative association between KAM during walking and joint space width in KOA patients. However, limited studies have investigated the relationship between foot posture and function and mechanical loads on the knee joint. Increased rearfoot eversion, rearfoot internal rotation, forefoot inversion, and foot progression angle (FPA) are associated with reduced KAM during walking [7, 8], and accumulating evidence indicates that KOA patients have abnormal foot posture and foot function, such as hallux valgus [9], pronated foot [10], flatfoot [11], and decreased toe grip strength (TGS) [12]. Notably, Gross et al. [11] found a significant association between flatfoot measured by footprint data and cartilage damage of the TFJ.

In clinical practice, foot evaluation is difficult using a three-dimensional (3D) motion analysis device (e.g., the Oxford Foot Model) owing to the time and cost involved. Many patients prefer to undergo physiotherapy and insole insertion after static foot evaluation that can be easily performed in clinical practice; hence, its relationship with TFJ loads must be determined. Rearfoot angle (RFA), navicular drop, and footprint as a uniplanar assessment of foot posture are often used [13]. In contrast, the Foot Posture Index (FPI) is a multi-planar tool that has gained popularity recently. Akaltun et al. [10] reported that foot posture assessed by FPI shows abnormalities such as more pronation or supination in patients with knee OA than healthy controls.

Many studies have used KAM as a surrogate marker of TFJ contact loading during activities, such as walking [6]. KAM is based on inverse dynamics, which only accounts for external parameters, i.e., ground reaction forces (GRF) and joint angles. Musculoskeletal modeling enables the calculation of muscle forces (MF) and joint contact forces and gives insights into the internal loading of body [5]. Notably, Lerner et al. [14] developed a model in OpenSim to accurately assess medial and lateral tibiofemoral contact force (CF) in a tibiofemoral implant study. This model uses subject-specific knee joint medial and lateral compartment contact locations and joint alignment to more accurately estimate medial and lateral tibiofemoral CF (MCF, LCF). Moreover, a systematic review investigating the correlation between tibiofemoral CFs and external joint moments found that the MCF and total tibiofemoral CF (TCF) in the first half of stance can be predicted using external joint moments. However, worse correlations were found for the peak in the second half of stance and LCF [15]. Therefore, while investigating the second peak in the stance phase and lateral knee joint loading, it is better to calculate tibiofemoral CF using musculoskeletal models.

Despite these findings, the biomechanical relationship between foot posture and function and tibiofemoral CF remains unclear. Therefore, this study aimed to clarify the association between tibiofemoral CF and foot posture and function during walking in KOA patients. We hypothesized that participants with pronated foot posture and function, such as excessive flatfoot, hallux valgus, and lower TGS, have increased tibiofemoral CF during walking.

## Methods

### Participants

This cross-sectional study included outpatients with KOA diagnosed by their treating physician in orthopedics at Kashiba Asahigaoka Hospital and healthy adults from participants in a health promotion project conducted by Koryo Town, Nara, Japan, and from the staff members of Kashiba Asahigaoka Hospital. All participants were recruited between March 2019 and January 2020.

The inclusion criteria for the participants with medial KOA were as follows: Age > 45 years and radiographic changes with Kellgren-Lawrence (KL) grade of 2 or higher in the medial tibiofemoral compartment. The exclusion criteria for the participants with medial KOA were as follows: A history of any other orthopedic injury in the lower extremities, neurological injury, rheumatoid arthritis, joint surgery in lower extremities, lateral KOA, or use of an assistive device. For control participants, the inclusion criteria were as follows: age > 45 years, walking independently without any ambulatory assistive device, and no pain in the knee joint, and we excluded those with any previous surgical treatment for the lower limbs or trunk or known neuromuscular or musculoskeletal pathologies.

### Gait analysis

Six VICON MX-F20 cameras (Vicon Metrics, Oxford, UK) and four floor-embedded force platforms (2 × 9281B, Kistler Instrument Corporation, Switzerland; 2 × AMTI BP400600, Advanced Mechanical Technology Inc., USA) were used to measure 3D kinematics and GRF with a sampling rate of 120 Hz, respectively. The plug-in-gait full body 39 markers set was used [16]. These markers were attached to each participant’s skin at the following anatomical landmarks: forehead, back of the head, shoulder, 7^th^ cervical vertebra (C7), 10^th^ thoracic vertebra, upper arm, elbow, forearm, wrist, finger, right back, anterior superior iliac spines (ASIS), posterior superior iliac spines (PSIS), thigh, lateral knee, tibia, lateral ankles, heels, and second metatarsal heads.

Participants were asked to walk at a self-selected speed, barefoot, along a flat 8-m walkway. Three successful walking trials were recorded. Stance phases during gait were determined using vertical GRF data. GRFs were low-pass filtered using 10-Hz cut-off frequencies for walking. The threshold value for the stance phase was a GRF of 25 N or more in the vertical direction. The walking speed was calculated from the distance of the right and left second metatarsal head marker positions during 6 steps and the time taken.

### Musculoskeletal model

A musculoskeletal model with 18 body segments and 92 muscle–tendon actuators in OpenSim was used to compute the tibiofemoral CF [14, 17]. The model can calculate accurate medial and lateral CFs, strictly reflecting the subject-specific knee joint alignment in the participant's lower extremity radiograph [14]. In this knee mechanism, the medial and lateral TFJs share all forces transmitted between the femur and tibia and resolve them as the medial and lateral tibiofemoral CFs required to balance the net reaction forces and frontal-plane moments across the TFJ. Correspondingly, the knee remains a single degree of freedom (DOF) joint with motion only in the sagittal plane. Therefore, the model includes a spline function on the knee axis, which takes into account the anterior–posterior translations [14, 17]. The model has only a single DOF for dorsi-/plantarflexion and a single axis for the ankle joint. For each participant, subject-specific models were created based on the approach reported by Lerner et al. [14]. In the KOA group, standing anatomical motion capture markers were supplemented with anteroposterior weight-bearing radiograph of the lower-extremity [14, 18]. The dimensions of each body segment in the model were scaled based on relative distances between pairs of markers obtained from a motion-capture system and the corresponding virtual marker locations in the model. Next, we modified each participant’s scaled model and created a subject-specific model by specifying their lower extremity alignment from the radiographs in KOA patients. An anteroposterior radiograph of the participant's lower-extremity was used to determine the subject-specific alignment for the musculoskeletal model. The tibiofemoral alignment was found by drawing lines connecting the hip, knee, and ankle joint centers, which were defined as the center of the femoral head, center of the femoral condyles, and midpoint of the medial and lateral margins of the ankle, respectively. The tibiofemoral alignment was adjusted only for the healthy adults using the marker information [19]. The model was then used to calculate joint angles and moments during walking trials using OpenSim inverse kinematics and inverse dynamics, respectively. MF were estimated using static optimization. Minimizing of activation squared was used as the static optimization criterion for the calculation of MF. These results were used to calculate the joint reaction force (JRF) in OpenSim.

### Foot assessment

The Foot Posture Index (FPI) was calculated in a relaxed standing position to assess foot posture based on a six-item standard protocol [20]. The FPI included the following: 1) talar head palpation, 2) supra and infra lateral malleolar curvature, 3) calcaneal frontal plane position, 4) prominence in the region of the talonavicular joint, 5) congruence of the medial longitudinal arch, and 6) abduction/adduction of the forefoot on the rear foot. Each item was scored on a scale of –2, –1, 0, + 1, and + 2 (0 for neutral, –2 for clear signs of supination, and + 2 for clear signs of pronation) [20, 21]. The total score ranged from –12 to + 12, with a larger positive value indicating a more pronated foot. For foot type identification, normative values with scores of ≥ 6 represent a pronated foot type; those with scores of 0–5 represent a neutral foot; and those with scores of ≤  − 1 represent a supinated foot [20, 21]. The inter-rater reliability for measuring FPI was high (ICC > 0.8) [22].

A force platform (WinFDM; Zebris, Isny im Allgäu, Germany) was used to measure static and dynamic footprint data. Static footprint data were recorded for 10 s with a participant standing on the platform with double limb support. Dynamic footprint data were collected during walking at a self-selected speed on the platform. The Staheli Arch Index (SAI), the ratio of the smallest width of the midfoot to the greatest width of the rearfoot [23], was calculated from footprint data. The value of the SAI increases with increasingly planus foot morphology and takes on a value of zero with cavus foot morphology. The widths were measured by ImageJ software [24]. A previous validity study indicated a moderately significant correlation between SAI measurements of foot morphology and radiological measurements of total foot morphology [23]. The average value of two measurements was used in the analysis. The inter-rater and intra-rater reliability for measuring the SAI was excellent (ICC > 0.9, respectively) [25].

The hallux valgus angle (HVA) and calcaneus inverted angle relative to the floor (CIA) were measured using a goniometer. HVA was the angle formed by the first metatarsal bone and the proximal phalanx of the hallux [26]. Janssen et al. [26] investigated the reliability, and concurrent validity of universal goniometer measurements of the HVA compared to those of radiographs and found a good intraclass correlation coefficient. CIA was defined as the angle formed by the line joining the bottom of the calcaneal tuberosity with the enthesis of the Achilles tendon and a line perpendicular to floor [27]. The reliability of CIA measurements was calculated by 2 testers for 20 participants. The inter-rater and intra-rater reliability for measuring the CIA was good (ICC: 0.92 and 0.86, respectively).

Navicular height (NH)/foot length was measured as the length from the floor to the top of the navicular tuberosity [28]. The measure of NH in a standing or sitting position demonstrated good intra- and inter-rater reliability [28].

Navicular drop test (ND-t) was recorded as the difference in NH between relaxed standing and sitting position [27]. ND-t had moderate intra- and inter-rater reliability [28].

TGS was measured using a toe grip dynamometer (T.K.K.3362; Takei Scientific Instruments Co., Ltd., Niigata, Japan) with participants sitting upright [29]. Participants gripped the grip bar with maximal effort for about 3 s. The average value of two measurements was used in the analysis. In this measurement protocol, substantial to almost perfect inter- and intra-rater reliability was found in people aged 60–79 years [29].

### Statistical analysis

Gait trial data were normalized by time to 0%–100% of the stance phase in the gait cycle. Each tibiofemoral CF, normalized by body weight (BW), was represented as a time series throughout the stance phase (SP). The first and second peaks of the TCF, MCF, and LCF were identified. The maximum tibiofemoral CF during 0%–50% of SP was defined as the first peak, and the maximum tibiofemoral CF during 51%–100% of SP was defined as the second peak.

Differences in demographic data, FPI, Standing SAI, Walking SAI, HVA, CIA, NH, ND-t, TGS, and gait speed between the groups were evaluated by a t-test. Sex differences were examined by chi-square test. Analysis of covariance (ANCOVA) was used to compare the tibiofemoral CFs between the KOA and Healthy groups adjusting for gait speed. For the relationship between foot posture or function and tibiofemoral CF, correlation coefficients of tibiofemoral CF with foot parameters that were significantly different between the groups were calculated. If a significant correlation between tibiofemoral CF and a foot parameter was found for the first peak, we then additionally performed a correlation between the MF at the peak and the foot parameter. The correlation between walking speed and tibiofemoral CF or foot parameters was further calculated. The Shapiro–Wilk test was used to determine the normal distributions. In normally distributed data, Pearson’s correlation tests were used. Partial correlations were used to assess relationship between tibiofemoral CFs and foot parameters while controlling for gait speed.

All statistical analyses were performed using SPSS Statistics for Windows, version 22.0 (IBM Corp, Tokyo, Japan). A *p*-value of < 0.05 was considered significant.

## Results

**Fig. 1:**
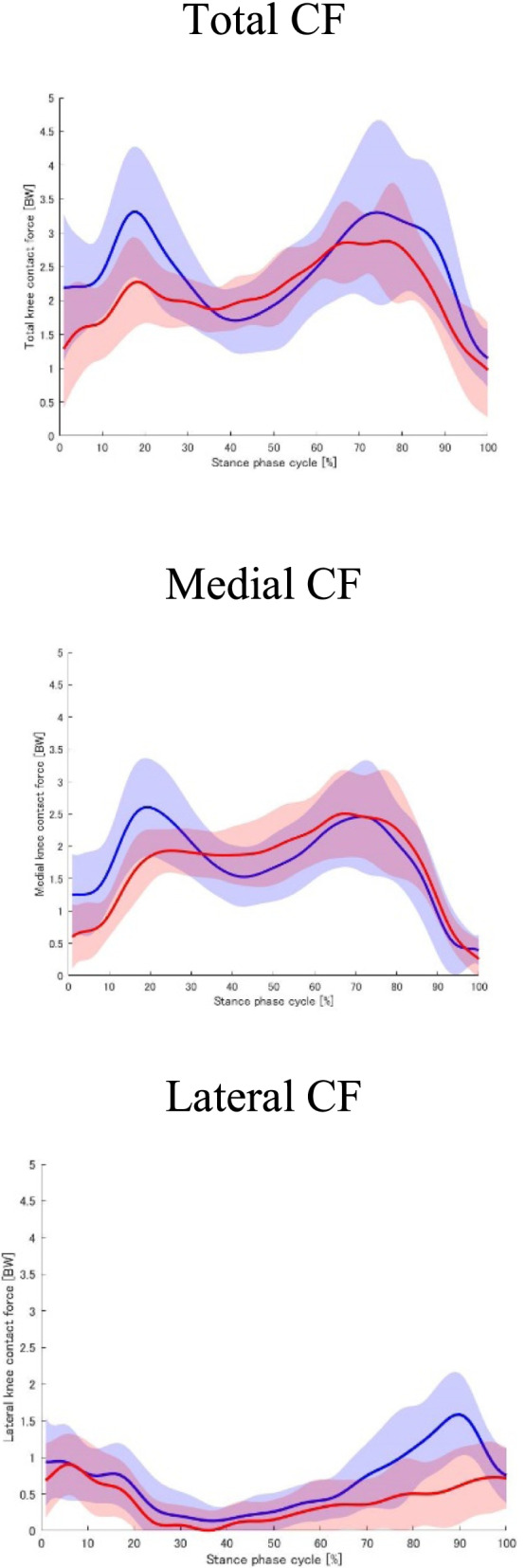
Force on the total, medial, and lateral tibiofemoral joint during the stance phase. Data are presented as mean ± standard deviation. Controls and patients with knee OA are presented as blue and red lines, respectively. CF, Tibiofemoral contact force

**Fig. 2:**
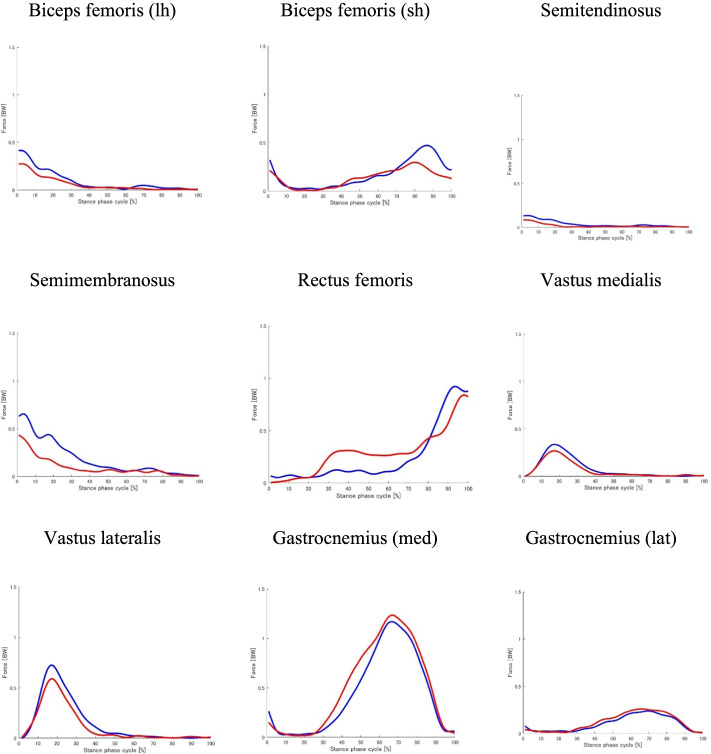
Muscle forces for knee-spanning muscles during the stance phase. Data are presented as mean ± standard deviation. Controls and patients with knee OA are presented as blue and red lines, respectively. Lh, long head. Sh, short head. Med, medialis. Lat, lateralis

Eighteen patients with medial KOA (4 men and 14 women; mean age: 60.17 ± 6.96 years) and 18 healthy adults (4 men and 14 women; mean age: 62.78 ± 8.58 years) were included in this study. Participant characteristics are shown in Table 1. There were no significant differences in age, height, and sex between KOA patients and controls. The participant weight was significantly higher in the KOA patient group than in the control group. KOA patients walked significantly slower than the controls. Foot assessment revealed no significant differences in the FPI, HVA, CIA, NH, and TGS between KOA patients and controls (Table 2). The Standing SAI, Walking SAI, and ND-t were significantly higher in KOA patients than in controls. Figure 1 shows the experimental results for the total tibiofemoral CF and medial and lateral tibiofemoral CF values. No differences were detected in the first and second peak medial, lateral, and total tibiofemoral CF values (Table 3). Figure 2 shows the experimental results for the MF of the knee-spanning muscle values. None of the parameters correlated with walking speed.

**Table 1:** Participant characteristics

	Controls (*n* = 18)	KOA (*n* = 18)	*p*-value
Age (years)	62.78 (8.58)	60.17 (6.96)	0.323
Height (cm)	157.83 (8.89)	161.44 (7.38)	0.194
Weight (kg)	54.33 (10.60)	65.97 (11.07)	0.003*
Female, n (%)	14 (78%)	14 (78%)	1.000
Walking Speed (m/s)	1.30 (0.15)	1.18 (0.13)	0.016*
Tibiofemoral alignment (deg)	177.35 (1.95)	180.28 (1.87)	
K/L grade, 2/3/4	N/A	11/ 7/ 0	

K/L grade, Kellgren and Lawrence grade. Values are presented as mean (SD). *P*-values were obtained from the independent t-test. *P < 0.05

**Table 2:** Foot characteristics

	Controls (*n* = 18)	KOA (*n* = 18)	*p*-value
FPI (point)	2.11 (2.42)	2.56 (2.91)	0.622
Standing SAI (mm/mm)	0.31 (0.26)	0.56 (0.24)	0.005**
Walking SAI (mm/mm)	0.46 (0.23)	0.62 (0.17)	0.020*
HVA (deg)	17.22 (7.36)	13.56 (7.14)	0.138
CIA (deg)	3.72 (2.19)	4.28 (3.69)	0.587
NH (mm/mm)	0.15 (0.02)	0.15 (0.02)	0.616
ND-t (mm)	0.46 (0.37)	0.72 (0.37)	0.042*
TGS (kg)	12.17 (4.06)	12.93 (5.09)	0.627

*KOA* Knee osteoarthritis, *FPI* Foot posture index, *SAI* Staheli Arch Index, *HVA* Hallux valgus angle, *CIA* Calcaneus inverted angle relative to the floor, *NH* Navicular height, *ND-t* Navicular drop test, *TGS* Toe grip strength. *P*-values were obtained from independent t-test. **p < 0.01, *p < 0.05

**Table 3:** Peak values of the first and second tibiofemoral CFs

	Controls (*n* = 18)	KOA (*n* = 18)	*p*-value
**Total** tibiofemoral **CF**			
First Peak (BW)	3.51 (0.91)	3.45 (0.87)	0.772
Second Peak (BW)	3.61 (1.37)	4.17 (1.11)	0.093
**Medial** tibiofemoral **CF**			
First Peak (BW)	2.71 (0.78)	2.65 (0.45)	0.773
Second Peak (BW)	2.58 (0.90)	3.09 (0.83)	0.072
**Lateral** tibiofemoral **CF**			
First Peak (BW)	1.24 (0.41)	1.60 (0.71)	0.069
Second Peak (BW)	1.79 (0.60)	1.43 (0.47)	0.060

All data are expressed as mean (SD). *CF* Contact force. *P*-values were obtained from independent t-test

**Fig. 3:**
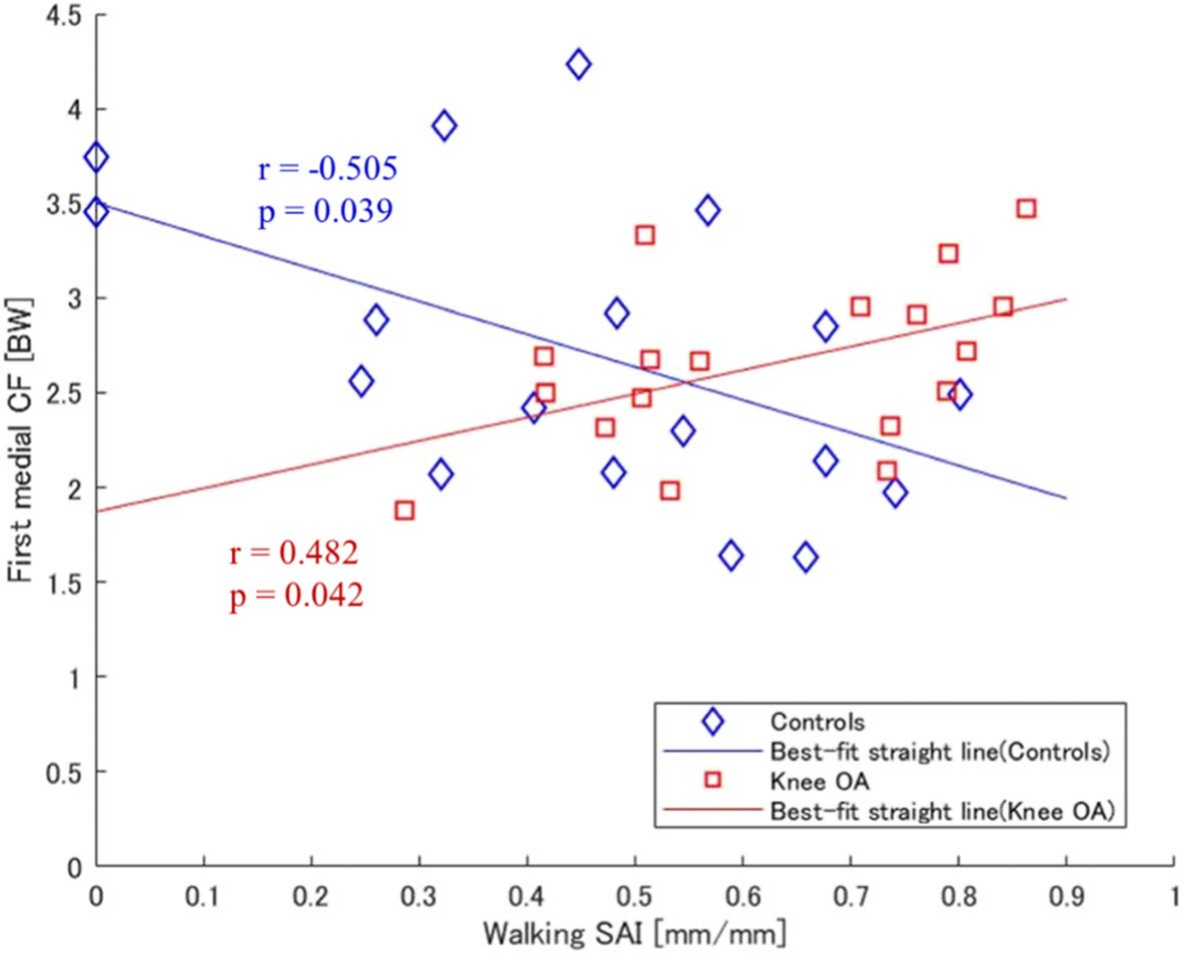
Scatter plot with the best fit line (solid line) for Partial correlations. The correlation analysis was performed between first medial CF and SAI for controls (diamonds) and knee OA (squares). CF, Tibiofemoral contact force. SAI, Staheli Arch Index

According to Partial correlations coefficient, the first peak MCF was significantly negatively correlated with SAI during walking (r, -0.505; p, 0.039) in KOA patients. The negative correlation indicates that for a people with a lower arch there is greater contact force in KOA. However, in healthy adults, the first peak MCF was significantly positively correlated with SAI during walking (r, 0.482; p, 0.042) (Fig. 3).

No significant correlation was found between the first and second peak values of the CF and Standing SAI and ND-t. No significant correlation was found between MF and SAI.

## Discussion

This cross-sectional study showed that flatfoot expressed as SAI during walking increases medial knee joint loading. To our knowledge, this study is the first to investigate the relationship between CF, calculated using the musculoskeletal model, and foot posture in patients with KOA. Many researchers have also reported a relationship between KOA and the foot parameters. Our research extends others’ work in this area.

Our results are consistent with those of previous musculoskeletal model-based studies [5, 30]. Figures 1 and 2 show the CF and MF values, respectively. These are similar to the waveforms in previous studies [5, 30]. The 12-month longitudinal observational study indicated that a medial knee joint loading was associated with an increased risk of medial knee OA structural progression [2]. Peak TCF of about 3–4 times BW was calculated. Tibiofemoral CFs showed no statistically significant differences between the groups in terms of first and second peaks. This result is consistent with that of a similar previous study [31]. Merireles et al. [31] reported that tibiofemoral CFs in patients with early KOA (KL ≤ 2) were not significantly different from those in healthy adults. Although the magnitudes of first and second peaks in the KOA group in this study have different trends compared to those in the study by Merireles et al. [31], the majority of patients had KL ≤ 2, and the relative peaks of tibiofemoral CFs between the groups were nonsignificant. In patients with KOA, strategies to reduce the loading on the knee joint during gait have been reported [32]. This may be due to the fact that the analysis in this study focused on static foot parameters and did not exclude the effects of other joints and other factors.

Our results showed that the correlations between the first peak MCF and Walking SAI were negative in KOA patients and positive in healthy controls. This means that the flatter the foot is in patients with KOA, the higher the MCF. In this study, of all of the tibiofemoral CFs, only the MCF was associated with Walking SAI, which are most affected by the moment arm length. KAM contributes to the tibiofemoral CFs. The moment arm is determined by the distance of GRF from the center of the knee joint. The position of the calcaneus during walking in healthy participants is different from that in patients with foot problems. The lateral shift of the calcaneus during walking in children with flatfoot results in a low first peak KAM because of the point of GRF application due to the calcaneal position [33]. In fact, shoe modifications produce a lateral shift in the point of GRF and reduce the first peak KAM in healthy adults [34]. Therefore, flat feet in healthy adults may bring the GRF closer to the center of the knee joint and have the potential to indirectly decrease tibiofemoral CF. KOA patients achieve a relatively flatter position of the foot while walking as compared to healthy adults. Further, previous research has shown that people with flatfoot show a high peak pressure for the medial foot area during walking [35]. In people with flatfoot, the center of pressure may be different during medial shift of the foot; as a result, the KAM moment arm is expected to increase. Moreover, the range of motion of the rearfoot is smaller in KOA patients than in healthy participants [36]. Therefore, the lateral shift of the calcaneus is unlikely to occur in KOA patients.

In healthy and KOA participants, only the MCF was associated with SAI, but the LCF was not significantly associated with SAI, probably due to the GRF and knee joint position.

MF showed no significant correlation with SAI during the first peak MCF. Muscles that do not pass through the ankle joint or foot are unlikely to be affected by SAI. The gastrocnemius is the only muscle that passes through the knee and ankle joints. However, previous studies showed no significant difference in muscle activity in the gastrocnemius during walking between people with flatfoot and healthy individuals [37]. These are similar to the results in the previous study [37], except for the gastrocnemius muscle, we could not draw a direct relationship as no muscle extends from the knee joint to the foot.

There was a significant difference in ND-t, Standing SAI, and Walking SAI between the healthy and KOA groups. During most weight-bearing activities, the posture and motion of the foot and knee are coupled within a closed kinematic chain, such as the movements in the horizontal plane and frontal plane [36]. However, despite the fact that Walking SAI is correlated, ND-t and Standing SAI had no correlation with tibiofemoral CF during walking. Controversies regarding static foot alignment reflecting a dynamic foot morphology exist [38, 39]. Additionally, the dynamic foot motion during walking in KOA patients differ from that in healthy adults [36]. Therefore, it is highly possible that the SAI evaluated during walking represented the tibiofemoral CF during walking. Furthermore, closed chain coupling may link excessively planus foot morphology to excessive internal rotation of the lower extremity [40]. However, in this study, only tibiofemoral CF was used, and foot, ankle, knee, or hip kinematics and rotational stress were not investigated.

This study had some limitations. First, the number of males was fewer than that of females. Future studies have to include an equal number of male and female participants. Additionally, the sample size used in this study was small, potentially leading to inconclusive or insignificant results. However, our results for the control and patients with KOA were comparable with those of previous studies. Second, the model had only a single DOF for flexion/extension and a single axis for the knee joint. Therefore, internal/external rotation and dynamic adduction/abduction movement during knee joint movement could not be considered. Third, the model of the ankle joint and midfoot motion used herein was for dorsiflexion and plantarflexion only. Therefore, this model did not accurately reflect the movement of the coronal plane motion in the subtalar and metatarsophalangeal joints. Along with them, the FPA has not been able to be considered. However, the GRF was actually assessed in this study; its relationship with the position of the center of gravity or knee joint was clarified using marker trajectory data. Fourth, we assumed the same 'normal' motor control/muscle recruitment strategy between healthy people and patients with OA in the musculoskeletal simulations. Unlike the EMG-informed model, it is likely that OA patient characteristics, including co-contraction, are not reflected by the model in this study. However, the use of an EMG-informed model in future studies may better characterize muscle contractions in healthy and OA patients. Furthermore, gait speed in patients with knee OA was much slower than that in controls. Thus, different results may be obtained in patients with knee OA who maintain gait speed. Finally, further studies are needed to investigate detailed relationships using more discriminating foot morphology indexes. Future studies directed toward investigating patients diagnosed with pathological foot positions or functions are warranted.

## Conclusions

The first peak medial tibiofemoral CF was significantly correlated with the SAI. The findings reveal the relationship between cavus foot and tibiofemoral CF during walking in patients with knee OA. This study suggests that patients with knee OA and flatfoot have excessive first MCF during walking.

## Supplementary Information

**Additional file 1.**

## Data Availability

The datasets used and analyzed during the current study available from the corresponding author on reasonable request.

## Abbreviations

3D: Three-dimensional
ASIS: Anterior superior iliac spines
BW: Body weight
CF: Contact force
CIA: Calcaneus inverted angle
DOF: Degree of freedom
FPI: Foot Posture Index
GRF: Ground reaction forces
HVA: Hallux valgus angle
KAM: Knee adduction moment
KL: Kellgren-Lawrence
KOA: Knee osteoarthritis
MF: Muscle forces
NH: Navicular height
ND-t: Navicular drop test
PSIS: Posterior superior iliac spines
RFA: Rearfoot angle
SAJ: Staheli Arch Index
SP: Stance phase
TFI: Tibiofemoral joint
TGS: Toe grip strength
